# Bounds of the Spectral Radius and the Nordhaus-Gaddum Type of the Graphs

**DOI:** 10.1155/2013/472956

**Published:** 2013-06-05

**Authors:** Tianfei Wang, Liping Jia, Feng Sun

**Affiliations:** School of Mathematics and Information Science, Leshan Normal University, Leshan 614004, China

## Abstract

The Laplacian spectra are the eigenvalues of Laplacian matrix *L*(*G*) = *D*(*G*) − *A*(*G*), where *D*(*G*) and *A*(*G*) are the diagonal matrix of vertex degrees and the adjacency matrix of a graph *G*, respectively, and the spectral radius of a graph *G* is the largest eigenvalue of *A*(*G*). The spectra of the graph and corresponding eigenvalues are closely linked to the molecular stability and related chemical properties. In quantum chemistry, spectral radius of a graph is the maximum energy level of molecules. Therefore, good upper bounds for the spectral radius are conducive to evaluate the energy of molecules. In this paper, we first give several sharp upper bounds on the adjacency spectral radius in terms of some invariants of graphs, such as the vertex degree, the average 2-degree, and the number of the triangles. Then, we give some numerical examples which indicate that the results are better than the mentioned upper bounds in some sense. Finally, an upper bound of the Nordhaus-Gaddum type is obtained for the sum of Laplacian spectral radius of a connected graph and its complement. Moreover, some examples are applied to illustrate that our result is valuable.

## 1. Introduction 

The graphs in this paper are simple and undirected. Let *G* be a simple graph with *n* vertices and *m* edges. For *v* ∈ *V*, denote by *d*_*v*_, *m*_*v*_, and *N*_*v*_ the degree of *v*, the average 2-degree of *v*, and the set of neighbors of *v*, respectively. Then *d*_*v*_ *m*_*v*_ is the 2-degree of *v*. Let Δ, Δ′, *δ*, and *δ*′ denote the maximum degree, second largest degree, minimum degree, and second smallest degree of vertices of *G*, respectively. Obviously, we have Δ′ < Δ and *δ*′ > *δ*. A graph is *d*-regular if Δ = *δ* = *d*.

The complement graph *G* ^*c*^ of *G* is the graph with the same set of vertices as *G*, where two distinct vertices are adjacent if and only if they are independent in *G*. The line graph *L*_*G*_ of *G* is defined by *V*(*L*_*G*_) = *E*(*G*), where any two vertices in *L*_*G*_ are adjacent if and only if they are adjacent as edges of *G*. 

Let *X* be a nonnegative square matrix. The spectral radius *ρ*(*X*) of *X* is the maximum eigenvalue of *X*. Denote by *B* the adjacency matrix of *L*_*G*_, then *ρ*(*B*) is the spectral radius of *B*. Let *D*(*G*) and *A*(*G*) denote the diagonal matrix of vertex degrees and the adjacency matrix of *G*, respectively. Then the matrix *L*(*G*) = *D*(*G*) − *A*(*G*) is called the Laplacian matrix of a graph *G*. Obviously, it is symmetric and positive semidefinite. Similarly, the quasi-Laplacian matrix is defined as *Q*(*G*) = *D*(*G*) + *A*(*G*), which is a nonnegative irreducible matrix. The largest eigenvalue of the Laplacian matrix, denoted by *μ*(*G*), is called the Laplacian spectral radius. The Laplacian eigenvalues of a graph are important in graph theory, because they have close relations to many graph invariants, including connectivity, isoperimetric number, diameter, and maximum cut. Particularly, good upper bounds for *μ*(*G*) are applied in many fields. For instance, it is used in theoretical chemistry, within the Heilbronner model, to determine the first ionization potential of alkanes, in combinatorial optimization to provide an upper bound on the size of the maximum cut in graph, in communication networks to provide a lower bound on the edge-forwarding index, and so forth. To learn more information on the applications of Laplacian spectral radius and other Laplacian eigenvalues of a graph, see references [1–4].

In the recent thirty years, the researchers obtained many good upper bounds for *μ*(*G*) [5–8]. These upper bounds improved the previous results constantly. In this paper, we focus on the bounds for the spectral radius of a graph, and the bound of Nordhaus-Gaddum type is also considered, which is the sum of Laplacian spectral radius of a connected graph *G* and its complement *G* ^*c*^.

At the end of this section, we introduce some lemmas which will be used later on.

**Lemma 1 (see [9])**

Let *M* = (*m*_*ij*_)_*n*×*n*_ be an irreducible nonnegative matrix with spectral radius *ρ*(*M*), and let *R*_*i*_(*M*) be the *i*th row sum of *M*; that is, *R*_*i*_(*M*) = ∑_*j*_ *m*_*ij*_. Then

(1) min⁡1≤i≤n Ri(M)≤ρ(M)≤max⁡1≤i≤n Ri(M).

Moreover, if the row sums of *M* are not all equal, then both inequalities are strict.

**Lemma 2 (see [10])**

Let *G* = [*V*, *E*] be a connected graph with *n* vertices; then

(2) ρ(G)≤12ρ(LG)+1.

The equality holds if and only if *G* is a regular graph.

This lemma gives a relation between the spectral radius of a graph and its line graph. Therefore, we can estimate the spectral radius of the adjacency matrix of graph by estimating that of its line graph.

**Lemma 3 (see [11])**

Let *B* be a real symmetric *n* × *n* matrix, and let *ρ*(*B*) be the largest eigenvalue of *B*. If *P*(*λ*) is a polynomial on *λ*, then

(3) min⁡v∈V Rv(P(B))≤P(ρ(B))≤max⁡v∈V Rv(P(B)).

Here *R*_*v*_(*P*(*B*)) is the *v*th row sum of matrix *P*(*B*). Moreover, if the row sums of *P*(*B*) are not all equal, then both inequalities are strict.

**Lemma 4 (see [11])**

Let *G* be a simple connected graph with *n* vertices and let *ρ*(*Q*) be the largest eigenvalue of the quasi-Laplacian matrix of graph *G*. Then

(4) μ(G)≤ρ(Q),

with equality holds if and only if *G* is a bipartite graph.

By these lemmas, we will give some improved upper bounds for the spectral radius and determine the corresponding extremal graphs.

This paper is organized as follows. In Section 2, we will give several sharp upper and lower bounds for the spectral radius of graphs and determine the extremal graphs which achieve these bounds. In Section 3, some bounds of Nordhaus-Gaddum type will be given. Furthermore, in Sections 2 and 3, we present some examples to illustrate that our results are better than all of the mentioned upper bounds in this paper, in some sense.

## 2. Bounds on the Spectral Radius

### 2.1. Previous Results

The eigenvalues of adjacency matrix of the graph have wide applications in many fields. For instance, it can be used to present the energy level of specific electrons. Specially, the spectral radius of a graph is the maximum energy level of molecules. Hence, good upper bound for the spectral radius helps to estimate the energy level of molecules [12–15]. Recently, there are some classic upper bounds for the spectral radius of graphs.

In the early time Cao [16] gave a bound as follows:

(5) ρ(G)≤2m−δ(n−1)+Δ(δ−1).

The equality holds if and only if *G* is regular graph or a star plus of *K*_2_, or a complete graph plus a regular graph with smaller degree of vertices.

Hu [17] obtained an upper bound with simple form as follows:

(6) ρ(G)≤2m−n−δ+2.

The equality holds if and only if *G* is *n* − 2 regular graph.

In 2005, Xu [18] proved that

(7) ρ(G)≤2m−n+1−(δ−1)(n−1−Δ).

The equality holds if and only if *G* is regular graph or a star graph.

Using the average 2-degree of the vertices, the rese-archers got more upper bounds. 

Cao's [16] another upper bound:

(8) ρ(G)≤max⁡u∈V(G)dumu.

The equality holds if and only if *G* is a regular graph or a semiregular bipartite graph.

Similarly, Abrham and Zhang [19] proved that

(9) ρ(G)≤max⁡uv∈E(G)dudv.

The equality holds if and only if *G* is a regular graph or a semiregular bipartite graph.

In recent years, Feng et al. [10] give some upper bounds for the spectral radius as follows:

(10) ρ(G)≤max⁡u∈V(G)du2+dumu2.

The equality holds if and only if *G* is regular graph.

(11) ρ(G)≤max⁡uv∈E(G)du(du+mu)+dv(dv+mv)2.

The equality holds if and only if *G* is regular graph.

(12) ρ(G)≤max⁡u∈V(G)du+dumu2.

The equality holds if and only if *G* is regular graph.

(13) ρ(G)≤max⁡uv∈E(G)du+dv+(du−dv)2+4mumv4.

The equality holds if and only if *G* is regular graph.

### 2.2. Main Results

All of these upper bounds mentioned in Section 2.1 are characterized by the degree and the average 2-degree of the vertices. Actually, we can also use other invariants of the graph to estimate the spectral radius. In the following, such an invariant will be introduced. 

**Figure 1:**
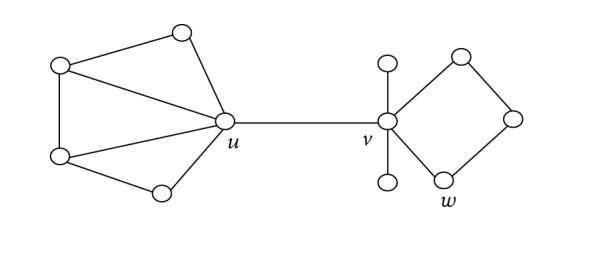
Graph with triangles.

In a graph, a circle with length 3 is called a triangle. If *u* is a triangle's vertex in a graph, then *u* is incident with this triangle. Denote by *T*_*u*_ the number of the triangles associated with the vertex *u*. For example, in Figure 1, we have *T*_*u*_ = 3 and *T*_*v*_ = *T*_*w*_= 0.

Let *N*_*u*_∩*N*_*v*_ be the set of the common adjacent points of vertex *u* and *v*; then |*N*_*u*_∩*N*_*v*_| present the cardinality of *N*_*u*_∩*N*_*v*_.

Now, some new and sharp upper and lower bounds for the spectral radius will be given.

**Theorem 5**

Let *G* be a simple connected graph with *n* vertices. Then

(14) ρ(G)≤max⁡uv∈E(G)du2mu+dv2mv−2(Tu+Tv)2(dudv−|Nu∩Nv|);

the equality holds if and only if *G* is a regular graph.

**Proof**

Let *K* = diag⁡(*d*_*u*_ *d*_*v*_ − |*N*_*u*_∩*N*_*v*_ | :*uv* ∈ *E*(*G*)) is a diagonal matrix and *B* is the adjacency matrix of the line graph. Denote *N* = *K* ^−1^ *BK*, then *N* and *B* have the same eigenvalues. Since *G* is a simple connected graph, it is easy to obtain that *N* is nonnegative and irreducible matrix. The (*uv*, *pq*)th entry of *N* is equal to

(15) {dpdq−|Np∩Nq|dudv−|Nu∩Nv|,pq~uv,0,else,

here *pq* ~ *uv* implies that *pq* and *uv* are adjacent in graph. Hence, the *uv*th row sum *R*_*uv*_(*N*) of *N* is

(16) ∑pq~uvdpdq−|Np∩Nq|dudv−|Nu∩Nv| =∑q~ududq+∑p~vdpdv−2dudvdudv−|Nu∩Nv|  −∑q~u|Nu∩Nq|  +∑p~v|Np∩Nv|−2|Nu∩Nv|dudv−|Nu∩Nv| =du2mu+dv2mv−2dudv−2(Tu+Tv)+2|Nu∩Nv|dudv−|Nu∩Nv| =du2mu+dv2mv−2(Tu+Tv)dudv−|Nu∩Nv|−2.

From Lemmas 1 and 2, we have

(17) ρ(G)≤12ρ(B)+1≤max⁡{12Ruv(N)+1:uv∈V(H)}.

It means that (14) holds and the equality in (14) holds if and only if *G* is a regular graph.

In a graph, let *α* and *β* represent the number of vertices with the maximum degree and minimum degree, respectively. Then, we get the following results.

**Theorem 6**

Let *G* be a simple connected graph with *n* vertices. If Δ ≤ min⁡{*n* − 1 − *β*, *n* − 1 − *α*}, then

(18) ρ(G)≤2m+Δ(δ′−1)−βδ−(n−1−β)δ′,

(19) ρ(G)≥2m+(Δ′−1)δ−αΔ−(n−1−α)Δ′;

the equality holds if and only if *G* is a regular graph.

**Proof**

Since *R*_*v*_(*A* ^2^) is exactly the number of walks of length 2 in *G* with a starting point *v*, thus

(20) $$\begin{array}{c} R_{v}\left(A^{2}\right)=\sum_{u\sim v}d_{u}=2m-d_{v}-\sum_{u≁v}d_{u}. \end{array}$$

Therefore, from Lemmas 1 and 3, if Δ ≤ *n* − 1 − *β*, we have *d*_*v*_ ≤ *n* − 1 − *β* for any *v* ∈ *V*(*G*). Then

(21) ρ(A2)≤max⁡v∈V(G)(2m−dv−∑u≁vdu)≤max⁡v∈V(G)(2m−dv−(βδ+(n−dv−1−β)δ′))=max⁡v∈V(G)(2m+(δ′−1)dv−βδ−(n−1−β)δ′)≤2m+Δ(δ′−1)−βδ−(n−1−β)δ′.

Hence, it is easy to obtain that (18) holds.

If equality in (18) holds, then all equalities in the above argument must hold. Thus, for all *v* ∈ *V*(*G*)

(22) $$\begin{array}{c} \sum_{u≁v}d_{u}=\beta \delta +\left(n-d_{v}-1-\beta \right)\delta^{\prime }. \end{array}$$

It means that *d*_*v*_ = *n* − 1 and *δ*′ = *δ*, or *d*_*u*_ = *δ* = *δ*′; this shows that the graph *G* is regular. Conversely, if *G* is *k*-regular, it is not difficult to check that *ρ*(*G*) attains the upper bound by direct calculation.

Similarly for the lower bound, if Δ ≤ *n* − 1 − *α*, we have

(23) ρ(A2)≥min⁡v∈V(G)(2m−dv−∑u≁vdu)≥min⁡v∈V(G)(2m−dv−(αΔ+(n−dv−1−α)Δ′))=min⁡v∈V(G)(2m+(Δ′−1)dv−αΔ−(n−1−α)Δ′)≥2m+(Δ′−1)δ−αΔ−(n−1−α)Δ′.

It means that (19) holds and the equality in (19) holds if and only if *G* is a regular graph by similar discussion.

**Theorem 7**

Let *G* be a simple connected graph with *n* vertices. If Δ ≤ *n* − 1 − *β*; then

(24) ρ(G)≤δ′−1+(δ′+1)2+8m−4β(δ−δ′)−4nδ′2;

the equality holds if and only if *G* is a regular graph.

**Proof**

According to the proof of Theorem 6, we have

(25) Rv(A2)=2m−dv−∑u≁vdu≤2m+(δ′−1)dv−βδ−(n−1−β)δ′.

Thus

(26) Rv(A2−(δ′−1)A)≤2m−βδ−(n−1−β)δ′.

From Lemma 3, we have

(27) ρ2(A)−(δ′−1)ρ(A)−2m+βδ+(n−1−β)δ′≤0.

Solving this quadratic inequality, we obtain that upper bound (24) holds.

If equality in (24) holds, then all equalities in the argument must hold. By the similar discussion of Theorem 6, the equality holds if and only if *G* is a regular graph.

### 2.3. Numerical Examples

**Figure 2:**
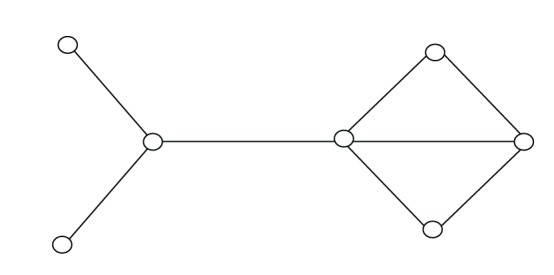
Graph of order 7.

**Figure 3:**
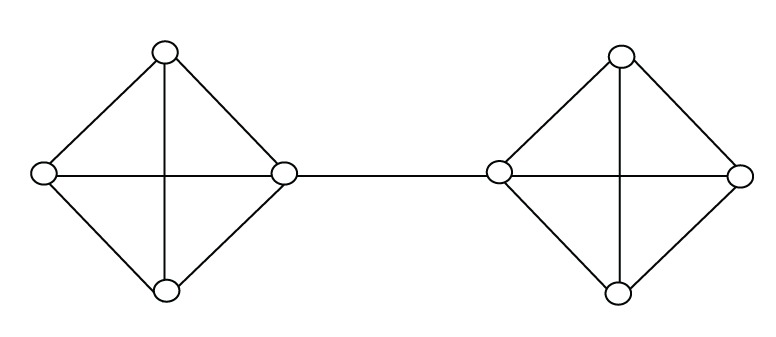
Graph of order 8.

In this section, we will present two graphs to illustrate that our some new bounds are better than other bounds in some sense. Let Figures 2 and 3 be graphs of orders 7 and 8.

The estimated value of each upper bound is listed in Table 1. Obviously, from Table 1, bound (24) is the best in all known upper bounds for Figure 2 and bound (14) is the best for Figure 3. Furthermore, bound (18) is the best except (13) and (24) for Figure 2. Hence, commonly, these upper bounds are incomparable.

## 3. Bounds of the Nordhaus-Gaddum Type

### 3.1. Previous Results

In this part, we mainly discuss the upper bounds on the sum of Laplacian spectral radius of a connected graph *G* and its complement *G* ^*c*^, which is called the upper bound of the Nordhaus-Gaddum type. For convenience, let

(28) σ(G)=μ(G)+μ(Gc).

The following are some classic upper bounds of Nordhaus-Gaddum type. The coarse bound *μ*(*G*) ≤ 2Δ easily implies the simplest upper bound on *σ*(*G*):

(29) σ(G)≤2(n−1)+2(Δ−δ).

In particular, if both *G* and *G* ^*c*^ are connected and irregular, Shi [20] gave a better upper bound as follows:

(30) σ(G)≤2(n−1−22n2−n)+2(Δ−δ).

Liu et al. [21] proved that

(31) σ(G)≤n−2+{(Δ−ω)2+n2+4(Δ−δ)(n−1)}1/2,

where *ω* = *n* − *δ* − 1.

Shi [20] gives another upper bound

(32) σ(G)≤2{(n−1)(2ω−δ)+(Δ+δ)2−Δ+δ}1/2.

To learn other bounds of the Nordhaus-Gaddum type, see references [22, 23]. In order to state the main result of this section, we first give an upper bound for the Laplacian spectral radius. 

### 3.2. Laplacian Spectral Radius

Here we give a new upper bound for the Laplacian spectral radius. For convenience, let

(33) f(m,Δ,δ)=((Δ−δ2−1)2+16m−2δ(4n−δ−2))1/2.

**Theorem 8**

Let *G* be a simple connected graph of order *n* with Δ and *δ*; then

(34) μ(G)≤Δ+(3/2)δ−1+f(m,Δ,δ)2,

with equality holds if and only if *G* is bipartite regular.

**Proof**

Let *K* = *Q* − *δE*; then *R*_*v*_(*K*) = 2*d*_*v*_ − *δ*, it means that 2*d*_*v*_ = *R*_*v*_(*K*) + *δ*. Considering the *v*th row sum of matrix *K* ^2^, we have

(35) Rv(K2)=Rv(Q2)−2δRv(Q)+δ2=2dv2+2∑u~vdu−4δdv+δ2=2dv2+2(2m−dv−∑u≁v,u≠vdu)−4δdv+δ2≤2dv2+2(2m−dv−(n−dv−1)δ)−4δdv+δ2=2dv2−2dv−2δdv+4m−2(n−1)δ+δ2=(2dv−δ)dv−(2+δ)dv+4m−2(n−1)δ+δ2≤ΔRv(K)−(2+δ)Rv(K)+δ2+4m−2(n−1)δ+δ2=(Δ−δ2−1)Rv(K)+4m−2nδ+δ+δ22.

This is equivalent to the following inequality:

(36) Rv(K2−(Δ−δ2−1)K)≤4m−2nδ+δ+δ22.

From Lemma 3, we obtain that

(37) ρ2(K)−(Δ−δ2−1)ρ(K)≤4m−2nδ+δ+δ22.

By simple calculation, we get the upper bound of the spectral radius of matrix *K* as follows:

(38) ρ(K)≤Δ−(δ/2)−12+((Δ−(δ/2)−1)2+16m−2δ(4n−δ−2))1/22.

Since *ρ*(*K*) = *ρ*(*Q*) − *δ*, therefore from Lemma 4 we obtain that the result (34) holds.

If the spectral radius *μ*(*G*) achieves the upper bound in (34), then each inequality in the above proof must be equal. This implies that Δ = *δ* for all *v* ∈ *V*(*G*), thus *G* is regular graph. From Lemma 4 again, *G* is regular bipartite graph.

Conversely, it is easy to verify that equality in (34) holds for regular bipartite graphs.

### 3.3. Bound of the Nordhaus-Gaddum Type

In this part, based on Theorem 8, an upper bound of Nordhaus-Gaddum type of Laplacian matrix will be given.

**Theorem 9**

Let *G* be a simple graph of order *n* with Δ and *δ*; then

(39) σ(G)≤5n−Δ+δ−9+2{2(2Δ−δ−2)2+8δ(2+δ)+(ω−Δ)(n+3Δ−3δ−5)+32nω−8π(3n+Δ−1)}1/24

here *ω* = *n* − *δ* − 1 and *π* = *n* − Δ − 1. Moreover, if both *G* and *G* ^*c*^ are connected, then the upper bound is strict.

**Proof**

According to the relation of a graph *G* and its complement, it is not difficult to obtain the invariants of *G* ^*c*^. Denote it by Δ(*G* ^*c*^) = *n* − *δ* − 1, *δ*(*G* ^*c*^) = *n* − Δ − 1, and *m*(*G* ^*c*^) = *C*_*n*_^2^ − *m*. From Theorem 8, we have

(40) μ(Gc)≤Δ(Gc)+(3/2)δ(Gc)−12 +f(m(Gc),Δ(Gc),δ(Gc))2.

Let

(41) g(m)=f(m,Δ,δ)+f(m(Gc),Δ(Gc),δ(Gc)).

Then the upper bound of the Nordhaus-Gaddum type of Laplacian matrix is

(42) $$\begin{array}{c} \sigma \left(G\right)=\mu \left(G\right)+\mu \left(G^{c}\right)\leq \frac{5n-\Delta +\delta -9+2g\left(m\right)}{4} \end{array}$$

since

(43) $$\begin{array}{c} g^{\prime }\left(m\right)=\frac{8}{f\left(m,\Delta ,\delta \right)}-\frac{8}{f\left(m\left(G^{c}\right),\Delta \left(G^{c}\right),\delta \left(G^{c}\right)\right)}. \end{array}$$

Obviously, *g*′(*m*) ≥ 0 holds if and only if the following inequality holds:

(44) $$\begin{array}{c} f\left(m,\Delta ,\delta \right)\leq f\left(C_{n}^{2}-m,n-\delta -1,n-\Delta -1\right). \end{array}$$

Let *m* be a variable; then solving this inequality, we have

(45) m≤(n−δ−Δ−1)(n−3δ+3Δ−5)+32n(n+δ−1)128−8δ(δ+2)−8(n−Δ−1)(3n+Δ−1)128=m∗.

Here, the symbol m* represents the right hand of the above inequality. Then we can assert that *g*(*m*) is an increasing function for *m* ≤ m*, and it implies that *g*(*m*) ≤ *g*(m*). Therefore, we have

(46) σ(G)≤5n−Δ+δ−9+2g(m∗)4=5n−Δ+δ−9+4f(m∗,Δ,δ)4.

Simplifying this expression by direct calculation, we prove that the result (39) is correct.

If equality in (39) holds, then each inequality in the above proof must be equality. From Theorem 8, we obtain that both *G* and *G* ^*c*^ are regular bipartite. But it is impossible for a connected graph, this implies that the Laplacian spectral radius of either *G* or *G* ^*c*^ fails to achieve its upper bound and so does the sum. Hence the inequality in (39) is strict.

### 3.4. Numerical Examples

**Figure 4:**
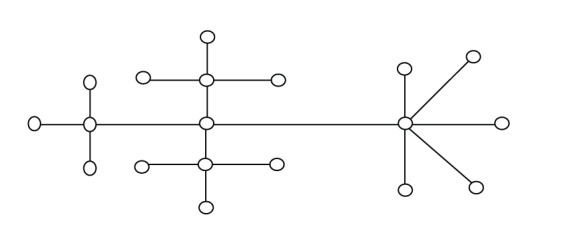
Graph of order 10.

In this section, we give some examples to illustrate that the new bound is better than other bounds for some graphs. Considering the graph of order 10 in Figure 4 and Figures 1–3, the estimated value of each upper bound of the Nordhaus-Gaddum type is given in Table 2.

Clearly, from Table 2, we can see that new bound (39) is the best in all known upper bounds for all figures mentioned in this paper.

## 4. Conclusion

From numerical examples of Sections 2 and 3, the estimated value of new upper bounds of the spectral radius and the Nordhaus-Gaddum type of graphs are the smallest in all known upper bounds for the graphs considered in these examples. It means that our results are better than the existing upper bounds in some sense.

## Figures and Tables

**Table 1:** Estimated value of each upper bound.

Upper bounds	Figure 2	Figure 3
Bound (5)	3.1623	4.1231
Bound (6)	3.1623	3.6056
Bound (7)	3.1623	3.6056
Bound (8)	3.1623	4.0000
Bound (9)	3.4641	3.8079
Bound (10)	3.6056	3.8079
Bound (11)	3.2787	3.6056
Bound (12)	3.5811	3.8028
Bound (13)	3.0650	3.6250
Bound (14)	3.5000	3.5000
Bound (18)	3.1623	4.0000
Bound (24)	3.0000	4.0000
Actual value	2.7321	3.3028

**Table 2:** Estimated value of each upper bound.

Upper bound	Figure 1	Figure 2	Figure 3	Figure 4
Bound (29)	28	18	16	46
Bound (30)	27.98	17.96	15.96	45.99
Bound (31)	26.23	16.05	15.59	46.02
Bound (32)	28.43	17.44	18.22	50.52
Bound (39)	25.84	15.88	15.52	44.97

## References

[1] Merris R (1994). Laplacian matrices of graphs: a survey. *Linear Algebra and Its Applications*.

[2] Mohar B, Harn G, Sabiussi G (1997). Some applications of Laplace eigenvalues of graphs. *Graph Symmetry*.

[3] Gutman I, Gineityte V, Lepović M, Petrović M (1999). The high-energy band in the photoelectron spectrum of alkanes and its dependence on molecular structure. *Journal of the Serbian Chemical Society*.

[4] Mohar B, Poljak S (1990). Eigenvalues and the max-cut problem. *Czechoslovak Mathematical Journal*.

[5] Wang TF (2007). Several sharp upper bounds for the largest laplacian eigenvalue of a graph. *Science in China*.

[6] Zhang XD (2004). Two sharp upper bounds for the Laplacian eigenvalues. *Linear Algebra and Its Applications*.

[7] Wang T, Yang J, Li B (2011). Improved upper bounds for the Laplacian spectral radius of a graph. *Electronic Journal of Combinatorics*.

[8] Wang TF, Li B (2010). New upper bounds for the laplacian spectral radius of graphs. *Journal of Sichuan Normal University*.

[9] Horn RA, Johnson CR (1985). *Matrix Analysis*.

[10] Feng L, Li Q, Zhang XD (2007). Some sharp upper bounds on the spectral radius of graphs. *Taiwanese Journal of Mathematics*.

[11] Li JS, Pan YL (2004). Upper bounds for the laplacian graph eigenvalues. *Acta Mathematica Sinica*.

[12] Shu JL, Hong Y, Wen RK (2002). A Sharp upper bound on the largest eigenvalue of the Laplacian matrix of a graph. *Linear Algebra and Its Applications*.

[13] Cvetkovic D, doob M, Sachs H (1997). *Spectral of Graphs: Theory and Applications*.

[14] Cvetkovic DM, Doob M, Gutman I, Yorgasev A (1988). *Recent Results in the Theory of Graph Spectra*.

[15] Biggs NL (1993). *Algebraic Graph Theory*.

[16] Cao DS (1998). Bounds on eigenvalues and chromatic number. *Linear Algebra and Its Applications*.

[17] Hu SB (2000). Upper bound on spectral Radius of graphs. *Journal of Hebei University*.

[18] Xu HJ (2005). Upper bound on spectral radius of graphs. *Journal of Jiamusi University*.

[19] Abrham B, Zhang XD (2001). on the spectral radius of graphs with cut vertices. *Journal of Combinatorial Thcory*.

[20] Shi L (2007). Bounds on the (Laplacian) spectral radius of graphs. *Linear Algebra and Its Applications*.

[21] Liu H, Lu M, Tian F (2004). On the Laplacian spectral radius of a graph. *Linear Algebra and Its Applications*.

[22] Hong Y, Shu JL (2000). A sharp upper bound for the spectral radius of the Nordhaus-Gaddum type. *Discrete Mathematics*.

[23] He S, Shu JL (2007). Ordering of trees with respect to their spectral radius of Nordhaus-Gaddum type. *Journal of Applied Mathematics*.

